# Using Twitter to Explore (un)Healthy Housing: Learning from the #Characterbuildings Campaign in New Zealand

**DOI:** 10.3390/ijerph14111424

**Published:** 2017-11-21

**Authors:** Elinor Chisholm, Kimberley O’Sullivan

**Affiliations:** He Kainga Oranga/Housing and Health Research Programme, Department of Public Health, University of Otago, Wellington, P.O. Box 7343, Wellington 6242, New Zealand; elinor.chisholm@otago.ac.nz

**Keywords:** twitter, housing, building quality, rental housing, building standards, public health, social media

## Abstract

While increasingly used for research, Twitter remains largely untapped as a source of data about housing. We explore the growth of social media and use of Twitter in health and social research, and question why housing researchers have avoided using Twitter to explore housing issues to date. We use the #characterbuildings campaign, initiated by an online media platform in New Zealand in 2014 to illustrate that Twitter can provide insights into housing as a public health and social problem. We find that Twitter users share details of problems with past and present homes on this public platform, and that this readily available data can contribute to the case for improving building quality as a means of promoting public health. Moreover, the way people responded to the request to share details about their housing experiences provides insight into how New Zealanders conceive of housing problems.

## 1. Introduction

The current iteration of the Internet, often described as “Web 2.0”, is characterized by the proliferation of user-generated content such as through social media sites, and which contrasts to the past, where most internet users were passive consumers of content [1]. Daily, millions of pieces of information are added to the Internet by users. The average daily time spent on social media worldwide was 118 min in 2016 [2]. In New Zealand, there are more than 370,000 Twitter users (about 7% of the population), with 10% of internet users having a Twitter account in 2015 [3]. Public figures, including advocacy groups use social media to help frame debates and draw in supporters [4]. In addition to news organizations, for example The New York Times with 39.4 million and The Guardian with 6.77 million followers respectively (of 13 September 2017), individual journalists are increasingly using Twitter to promote their work, break stories and connect with sources and their audiences in a less formal format [5].

Hashtags—words or phrases preceded by the “#” pound symbol—group tweets on the same topic together and enable users to take part in a wider conversation [6]. Hashtags are used by media to either generate or contribute to ongoing stories. The reverse is also evident, with comments from Facebook and Twitter increasingly used in place of in on-the-street public comments in media reporting [7,8,9]. Trending hashtags can also influence whether issues become stories within the mainstream media, in some cases, “setting mainstream news agendas and shaping the public debate” [10].

Researchers have been quick to take advantage of the vast amount of content generated by Web 2.0. In particular, social media is a useful way of capturing youth and younger-adult voices. Twitter is especially useful for this, given that users tend to be younger than other social media users [11]. Twitter allows people to post tweets—at the time of writing, limited to 140 characters—to followers, who may “retweet” them to their own followers. While other social networking sites have more users—Facebook has 2.01 billion monthly active users as of 30 June 2017 [12], as opposed to Twitter’s 328 million at 12 August 2017 [13]—Twitter has advantages to researchers over other social networking sites, in that its data is open, free, and around 90% of users’ accounts are publicly available in real time. In comparison to traditional polls or surveys, which are costly and time-intensive, people on Twitter are “freely volunteering information on a virtually unlimited number of topics” ([14], p. 16). In contrast to in-depth interviews, Twitter allows people to respond at their own convenience—which may be quite important for young people and those with caring responsibilities, who are often time-poor—and reduces power imbalances that are inherent in conducting interviews, even where researchers are careful to minimize these. Reviewing 380 scholarly publications which draw on Twitter-based research, across disciplines including computer science, business, economics, education, medicine, political science, and sociology, Zimmer and Proferes conclude that Twitter is a “valuable resource for tapping into the *zeitgeist* of the internet, its users, and often beyond” [15]. Researchers analyze Twitter content to better understand public opinion, sentiments and personality, online political phenomena such as activist or political campaigns or networks and communities, as well as real time events such as natural disasters or the spread of disease [15]. Twitter research may be based on qualitative content analysis of a select group of users or a particular topic, such as research on what health concerns people report [16]. It can also be based on large scale quantitative analysis of trends or topics—from how a political idea spreads, how earthquakes are experienced, or how sentiments expressed on Twitter correlate to market performance or to geography [15]. Researchers have also taken advantage of the way Twitter users group tweets under particular hashtags, looking at how #iranelection connected protesters and international supporters after Iran’s 2009 elections [16], how #whyistayed transformed online personal expressions of experience of domestic violence to collective political action [17], and how #IHMayDay assisted dialogue, advocacy and exchange about the social and emotional wellbeing of Aboriginal and Torres Strait Islander peoples [18].

Despite the potential of Twitter as a research tool, the use of Twitter as a resource for housing researchers has been limited. Taylor and Narayan analyzed one homeless person’s tweets and blog posts over the course of two years, finding that the online networks provided the user with valuable information, and opportunities to create and sustain social ties. Blogging and tweeting also allowed the user to “make sense of life’s struggles” and was a valuable source of information for researchers on a homeless person’s life [19]. Farrell’s auto-ethnographic account reflects on how using Twitter had supported a law center focused on homeless people’s rights to influence parliamentarians, built relationships with other advocates, and influence media framing of homelessness issues [20]. The range of uses of Twitter-based analysis in other fields of study shows that housing researchers could clearly benefit from use of this tool. For example, large-scale quantitative analysis, potentially drawing on geocoding, could give insight into the prevalence and distribution of particular housing issues or of health conditions related to housing. Qualitative housing researchers could analyze samples of tweets to learn about issues of interest such as affordability, security, mobility, tenancy law, housing-related activism, and fuel poverty. Here we provide an example of this type of empirical qualitative research: a content analysis of tweets that used #characterbuildings, a hashtag used to draw attention to New Zealand’s poor rental housing quality. 

Poor housing conditions, including insufficient heating and insulation, poor ventilation, and leaks and gaps in roofs, floor, and walls, are widespread in the New Zealand rental housing sector [21]. Inadequate housing has important implications for health [22]. For example, structural deficiencies contribute to injuries, especially due to falls [23,24]; mold, dampness and cold exacerbate asthma and other respiratory symptoms [25,26], and cold indoor environments cause increased blood pressure, particularly for those with indications of cardiovascular disease [27]. 

The hashtag #characterbuildings was created by the online media organization “*The Wireless*” on 22 September 2014. It was tweeted alongside a link to a story [28] about an initiative by a group of university students encouraging tenants to contribute to an online database called RateMyFlat in Dunedin, a city in the South of the South Island of New Zealand notorious for housing problems [29]. Entitled “Rate My Flat to make homes less ‘character building’”, the article also drew attention to a potential solution—a Warrant of Fitness, or minimum standards that houses must meet before being rented out [28]. Tweeting to followers, a journalist at *The Wireless* initiated the hashtag with the following tweet to followers, which included a link to the story [28]: “On @TheWirelessNZ we’re talking about flats not fit for habitation. Share your horror stories with #characterbuildings”. While the hashtag #characterbuildings does not refer specifically to rental properties, the article and the reference to “flats” made it clear that rental housing was the focus. (In New Zealand “flats” refers to houses as well as apartments, and is usually only used to describe rental properties.) In tweeting the hashtag, *The Wireless* encouraged people to talk about their flats or their flatting experiences. *The Wireless* stimulated use of the hashtag through tweeting the story and retweeting some of the contributed tweets using the outlet’s @TheWirelessNZ handle later that day, and TrendsMap tweeted that the hashtag was trending in New Zealand. Followers of *The Wireless’s* Twitter account quickly retweeted tweets that used the hashtag, thereby broadcasting the idea to a much wider audience. 

Therefore, this paper explores seeks to address the two broader questions: is existing Twitter data useful for exploring experiences of poor housing quality; and could Twitter data be useful for housing research generally? Through focusing on tweets that used #characterbuildings—a self-selecting convenience sample that isolates tweets about a phenomenon of major research and public concern—we also answer the specific research question: how do people responding to the #characterbuildings hashtag portray rental housing problems in New Zealand on Twitter? 

## 2. Materials and Methods

Data was collected on 10 May 2017. Twitter collects all tweets using specific hashtags under specific webpages. We put the tweets on the relevant webpage [30] in reverse-chronological order (by clicking on “latest” on this webpage). The earliest use of this hashtag on Twitter was 27 October 2011 and the latest use was 21 August 2016. (The four tweets posted prior to the use of the hashtag via *The Wireless* were about historic buildings or the building of character, rather than rental housing quality.) We copied and pasted all tweets, whether or not they were connected to *The Wireless’s* use of the hashtag, and including photos, that used this hashtag onto a document for subsequent analysis. The first author reviewed the tweets in order to develop a coding scheme. This scheme was reviewed and agreed by the second author, with one code (structural) refined after discussion to include mentions of insulation. Subsequently, and following the approach of Lee and colleagues (2011), each author conducted separate content analyses of each tweet on a preliminary sample of the first 60 tweets, arranged chronologically by posting time [31]. This approach to content analysis ensures consistent coding [32]. Tweets were ascribed to a code or codes, and a note of the content of photos was also made. Some tweets covered several areas and were given several codes accordingly. We compared our results to create a coding scheme (see Table 1, columns 1–3). Areas of disagreement were resolved on a case-by-case basis. We then used this coding scheme to code the remainder of the sample, both reading and agreeing to the final coding of the entire sample before analysis. 

For the analysis, we broadly adhere to qualitative description as put forward by Sandelowski [33,34], which results in a data-near analysis, useful for projects in which the goal is summary description of a phenomenon and policy-relevant discussion. This technique uses thematic qualitative coding as well as quantitizing or counting. Through quantitizing we provide proportions to indicate the relative importance of the different codes within the sample. Confidence intervals for the proportions (Table 1) were calculated with Wilson score using OpenEpi [35].

On consideration of ethics in when using Twitter data in social research (for a useful discussion of this see [15]), we present the results of this analysis here without using identifying Twitter handles. While these tweets are already present in the public domain and furthermore, were tweeted originally in response to a media organization implying a broader readership than their usual followers, it is our belief that inclusion of unidentified tweets is sufficient for the purpose of this discussion. We provide only textual descriptions of photos here for the same reason. 

## 3. Results

As at 10 May 2017, when coding for this project took place, 327 tweets had used the hashtag #characterbuildings. The majority of these (312) were tweeted within the first three days after *The Wireless* first used the hashtag. Thirty tweets were excluded as they met predetermined exclusion criteria. Excluded tweets referred to housing outside New Zealand or to impermanent housing (such as camping huts). Tweets were also excluded if they were satirical (for example, making fun of people living in luxury homes), or off-topic (for example, referring to property sales). This was to ensure our research shared the same focus as the hashtag: New Zealand rental housing quality. All other tweets using the hashtag were included. A total of 297 tweets were therefore coded for this analysis. Despite the character limit, tweets often discussed more than one of the issues coded for in Table 1, as illustrated by some of the examples provided, therefore the proportions expressed do not sum to 100. 

We discuss selected categories below, describing issues that represent most commonly tweeted, as well as unusual findings arising from the data. We relate these to what is already known about the key problems in New Zealand housing. It is helpful to note at the outset that it was not explicit that all of these tweets described rentals, but many tweeters indicated that they did not own the dwelling(s) to which they referred.

### 3.1. Structural Issues 

Over a third (34.7%) of #characterbuildings tweets described structural deficiencies with the dwelling including issues with floors (9.1%), the roof or ceiling (7.7%), or walls (8.4%), as well as draughts, holes, gaps, and pipes. Five noted structural problems with windows, a further five with balconies, earthquake damage was mentioned by two tweeters, one had a problem with a door and another with the foundations. Many of the structural issues described posed a significant risk to safety, such as holes in walls (2.4%), or floors (5.7%). A photo showed weeds (at least half a meter high) growing up through cracks in the floor in the corner of a living room. One tweeter reported that “my foot went through the rotten wood in front of the shower that had been hidden by lino”, and another that “Lino was the only thing holding up the bathroom floor. I was 6 months pregnant when we left—hadn’t showered in 3 weeks”. Two further tweets reported showers falling through the floor, and another a refrigerator that “literally fell through the floor”. One tweeter related that “my sister’s flat had a hole in the floor of the upstairs bathroom w a bit of 4 × 2 to cross it. Had to yell to warn ppl [people] below”. Perhaps the most extreme case describing structural issues was illustrated by this tweet: “Bath fell through floor. Bather unhurt. Hedgehog came into kitchen regularly, through hole in wall. Ate cat’s food”. 

Holes in the ceiling or roof, or leaks were described by 24 (8.1%) of the tweets and these often suggested significant water ingress or resulting damage, for example “Flatmate 1: The ceiling has collapsed onto my bed & now it is raining inside. Flatmates 2–6: Unsurprised”. One tweet described a leak from a toilet into a bedroom below, and three tweets described leaks that ran through light fittings. 

### 3.2. Landlord 

Of the 37 tweets that mentioned the landlord or property manager, 21 (56.8% of “Landlord” tweets) described poor maintenance that tenants often implied they had waited for. When maintenance was carried out it was mostly described as either “remedies” as one tweeter put it—Band-Aid solutions to a larger structural issue as described above, or a justification for increasing rent. For example, one tweet described a situation where “Plaster ceiling over fireplace collapsed. LL [Landlord] fixed with customwood and screws.” Some described maintenance requests being refused, even where problems were clearly a threat to health and safety, for instance, “Rain through light fittings indoors when it rained outdoors. Landlord wouldn’t do shit to fix”. In another case, there was a feeling of deception relayed along with an unreasonable delay: “Nine months no stove, they sent the same sparky to look at it, said the LL [landlord] already told him not to actually fix it”. Other tweets described clearly unreasonable landlord behavior, demonstrated by a landlord that asked to “put some lights and hydroponic equipment in the ceiling cavity in exchange for free power” and another who “occasionally stumbled in drunk thinking he still lived there”. Further tweets described inconvenient (but not illegal) landlord behaviors such as requiring tenants to store furniture or pointing out care and maintenance tasks that are associated with living in, rather than owning a property: for instance “told to open more windows to solve the condensation problem, most of the windows do not open due to quake damage”. Other tweets described the landlord threatening to raise the rent in order to carry out maintenance, or raising the rent after carrying out maintenance. These requirements and “reminders” and threats to rental security associated with requesting maintenance undermine renters’ notions of “home”, which are important for mental health and wellbeing [36]. These tweets add context to some of the previous themes: while tenants are aware of and unhappy with their housing problems, their capacity to take action on this is limited. While New Zealand renters do have rights to housing that meets certain standards, tenancies are relatively insecure. Attempts to enforce rights through contacting the landlord or the Tenancy Tribunal feels risky to tenants, given the lack of alternate housing options [37,38].

### 3.3. Facilities 

Lack of facilities or defects in facilities ranging from supplied curtains, carpets, or furnishings, to heating, plumbing, or electrics were mentioned in 33 tweets (11.1%). People shared stories of ineffective heating: “Fire is going but still need to be well wrapped up.” They noted problems with the bathroom: “The floor of the downstairs shower is rusted through in places”. They lived with faulty and dangerous wiring: “the lights dimmed when the toilet flushed”; “Plain wires spliced into the garage circuit, and ran unshielded under the back lawn. Used to short when it rained”. Poor plumbing was also described which was either inconvenient—for instance “Plumbing system was shit, header tank would shake house from excess water pressure till kitchen tap was run”—or eventually led to greater structural problems, with one person sharing a photo of a large piece of concrete that had fallen out of the garage ceiling onto the back of the roof of the car, showing an exposed leaking pipe which appeared to have caused cracks in the concrete ceiling. 

### 3.4. Cold

Twenty-six tweets (8.8%) were coded “cold”. Of these almost one third (31%) included tweets about structural problems in the home, including gaps in the walls and draughts, while five mentioned damp. A number of the tweets offered proof of the coldness of homes, for example, three people noted shampoo freezing in its bottle, one recalled another waking to ice on their blanket, and another how the drips from the bath tap froze to a stalactite. One recalled their hamster hibernating in winter. Three noted ice on the inside of windows or walls: one asked ironically, “Condensation frozen inside the windows is normal right?”. Four people shared how the air temperature was so cold that they could see their breath—a surprisingly small 1.3% of the overall sample, given other local research has found that more than a quarter of young New Zealanders were able to see their breath condensing inside their home on at least two occasions during the winter [39]. The same is true of 60.4% of New Zealand adults using prepayment metering to pay for electricity [40]. The common nature of this scenario in Wellington suburbs is illustrated by the following tweet: “Boasted to IslandBay sis my Kelburn flat had “visible breath” in bathroom: “SFW [So fucking what?]—we have visible breath IN THE BEDROOM”.

Other people shared their strategies for keeping warm in their homes: four noted that they sleeping with hats, balaclavas and pillowcases on their heads; as one noted “it is like camping”. One person noted falling asleep against a heater, while another recalled that they “studied wrapped in a blanket, an electric blanket plugged in and wrapped around that, in a sleeping bag”. 

### 3.5. Damp and Mould

The issues of damp and mould are closely related, particularly in New Zealand housing which is chronically underheated and faces a temperate but relatively wet climate in many of the most populated areas. New Zealand also has a large proportion of dwellings (as well as other buildings, for example public school buildings [41]) that have been subject to “leaky home syndrome” where inadequate regulation for building standards in the 1990s allowed untreated timber framing to be used, resulting in rotting, damp and mould problems [42]. One in five tweets within the sample described either damp or mould and we discuss them together here.

Twenty-eight tweets (9.4%) were coded as damp. Of these tweets 39% also mentioned mould, and 21% structural issues. Almost one third of tweets categorised as “damp” (32%) noted wet or condensated walls, a number of these with walls so wet the water was running or dripping; one person noted that the constant dampness had made the walls “malleable”. Tweeters also described damage caused by dampness. One noted that the he or she was “Currently in the process of ruining all of my posters just by putting them on walls because it is so damp”; another recalled a house which “had rising damp so bad if you left a book on the floor it was ruined by morning”. People shared strategies for protecting their belongings from dampness: “We hung plastic rubbish bags on hangers in the wardrobe to try and keep water from the wall soaking into our clothes”; “So damp I covered my bed with tarp [tarpaulin]”. 

Thirty-one tweets (10.4%) were coded as mould; almost one third of these (32%) described fungi, including mushrooms and toadstools, growing inside the house. People sharing how mould overtook their belongings, creating “blue-suede shoes”. One noted that he “Had to throw out an entire wardrobe of clothes after one winter in Wellington as all were covered in mould”. The health effects of living with mould should not be understated, with recent local research identifying statistically significant associations between the onset of childhood wheezing and both visible mould, and mould odour [25]. 

### 3.6. Pests

Pest problems were identified by 43 tweets (14.5%), with rodents identified in almost half of these (46.5%) as illustrated by this storied account: “As we watched, a mouse nibbled a hole in the kitchen ceiling and used it as a longdrop. I still live there”. 

Slugs are an issue associated both with structural defects (slugs can enter houses through cracks in the floor and walls) and damp (slugs prefer damp conditions) [43]. Ten tweets, or 3.4% of the total sample, and 23% of those discussing pests, reported living with slugs in their home. One recalled “the 3rd flat I moved to had slugs coming through the walls & we were evicted when we pointed this out”. On a related issue, another person noted “my old flat everytime it rained worms would come out of the laundry floor”. Thirteen tweets (4.4%) discussed other pests. 

### 3.7. Health

Surprisingly, as the ill-health effects of living in poor quality housing is a widely known and discussed issue in New Zealand, few tweets (2.4%) discussed ill-health relating to living in #characterbuildings. Six tweets mentioned respiratory health issues associated with their housing. Two people described chronic sinus issues, one stating “I breathed out of one nostril for the entire time I lived in Aro Valley”, a shady Wellington neighborhood. Two people described cleaning black mold in their homes; after this, one had to start using an inhaler for asthma symptoms, and the other reported the start of allergic symptoms. One noted that leaving one home was important to their health: “The underside of my mattress was green with mold when I moved out. My ongoing cough cleared up fairly quick after that.” Another described the low rents at their house, noting dryly “We paid the rest in respiratory illnesses”.

### 3.8. Cost

This trade-off between affordability and comfort was highlighted in 16 tweets: fifteen discussed the cost of rent, bills or upgrades in the context of the quality of the home. In some cases, people were aware of the shortcomings of their home but had no superior options. As one explained “we do not have any insulation and only have brick walls inside but it was the only place we could afford in town”. This dilemma is implicit in other tweets where people shared the cost of rent and a description of their home, for example: “The roof of my bathroom is corrugated plastic. We’re talking a 56 m^2^ $420 a week cottage here, friends.” People continued to live in substandard homes to avoid rent increases: “Yes that is a tree growing in the hole in the bathroom floor. LL said he’d put rent up for maintenance”. Maintenance was also used as a justification for increasing rent: “Landlord put the rent up when I asked them to fix the waterfall that appeared in my room during heavy rain”. Living in a substandard home entailed costs. One person listed the cost of power bills, and the cost of installing curtains and heating. Another recalled: “Ran a dehumidifier 24/7 (luckily I could afford to). If it stopped the condensation on the windows soaked walls & carpet.” 

For some, living in poor quality housing was worth it for the location. For example, one person recalled: “Roof leaked, mold, more pressure from a toddler pissing on me than the shower. Fuck it, 80 p/w [per week] in Mission Bay!” One compared their own experience as a student living in substandard dwelling in a now-gentrified inner-city suburb to the experience of students today: “used to live in shithaus flats but at least could walk to uni [university]. Now they bus from miles away. A prayer for old #greylynn”. 

### 3.9. Comments

Forty-three tweets did not share personal stories, but instead commented on the problem of poor quality housing in New Zealand. Some of these were by *The Wireless* and supportive commentators, drawing people’s attention to the hashtag. Some summed up key themes by users of the hashtag or reflected on the poor state of housing in New Zealand: “Man, New Zealander’s live in shit pits”. Other tweets had a nostalgic quality—“It is amazing what we put up with when we’re young & poor!”—which may tap into an idea of living stoically in a poor quality rental home as a fundamental rite of passage in the New Zealand student experience [44]. Another person seemed to wistfully reference this in their comment: “Feeling left out with all these #characterbuildings tales”. However, this idea is notably distinct from reality, where many New Zealanders continue to live in poor quality housing far beyond their youth [45]. Others responded in sympathy to individual tweets, or noted that their own negatives experiences in rental housing paled in comparison to those tweeted out: “Man, all the #characterbuildings tweets make me feel my flat with just bathroom mushrooms & a backyard of an active building site was fine”. This sentiment reflects a conclusion by Witten et al. that: “it was common for tenants to say they felt “lucky” to have had mostly all good landlords or rental experiences” ([46], p. 55).

## 4. Discussion

People using #characterbuildings provided vivid details of their housing experiences. Many of these reflected some of the key problems identified in New Zealand rental housing by housing and general population surveys. For example, in the BRANZ 2015 survey, 31% of rental dwellings were rated by assessors as damp, 56% of homes had some visible sign of mold, 32% of dwellings were poorly maintained, and 18% of dwellings had full-depth holes or cracks in the wall cladding [21]. Yet tweeters not only describe housing problems, but on relating their experiences provide vivid storied examples of the extent to which structural and other problems impacted on their lives: of watching mouse droppings fall from a hole in the ceiling, or vegetables roll away down a sloping floor; of having a book disintegrate overnight due to the damp floor, or a hamster hibernate from the cold. The tweets relate renters’ strategies for dealing with housing problems: sleeping with a woolen hat on, a tarpaulin on the bed, permanently running a dehumidifier, and separating clothes with plastic bags to prevent mold growth. These kinds of coping strategies for dealing with cold and damp are similar to those commonly reported by the fuel poor, both in New Zealand [47] and internationally, for example in Australia [48], the United Kingdom [49,50,51], parts of Europe [52,53] and Northern America [54]. The tweets also indicate why people continue to live in poor housing conditions: they cannot afford to move to superior housing, and they are aware that requesting maintenance may cause the rent to rise above their capacity to pay. 

A number of tweets were funny or ironic. People reported having to mow the living room floor (due to weeds growing on damp carpet or through cracks between the walls and floor), or missing the home that was so cold that the shampoo routinely froze. They described the hole in the bedroom wall as providing a convenient view of the garden, or characterized draughty homes as having “indoor-outdoor flow”, and the household slugs as a “spirit animal”. People may have used humor as a way of entertaining and attracting Twitter followers. There is evidence to suggest that use of humor on Twitter among journalists is used to connect to people, often by sharing personal anecdotes [5]. The tweets also suggest that people may use humor as a coping mechanism to reduce stress associated with dealing with housing problems [55]. As such, the tweets collected under #characterbuildings prove to be a useful resource for understanding people’s housing experiences, adding humanity to the statistics. 

The data also point towards housing problems that may be under-researched. Ten tweets referred to issues with slugs in the home; this is also a problem recently covered in the New Zealand media. There are a number of native and non-native slugs in New Zealand. The literature suggests that garden slugs can spread disease. Despite a thorough literature search of seven databases, we could find no research on the extent of the problem of slug infestations in housing, or of any health risks that this may pose. Ten tweets referred to mushrooms or toadstools growing inside. Media articles suggest people are afraid of removing mushrooms due to fear of poison. Yet similarly, there is little guidance on the precautions people should take when removing mushrooms. While the New Zealand government provides guidance to tenants on reducing moisture, mold and cold in their homes, no guidance is provided on how people should respond to mushrooms or slugs in their homes. 

While tweets such as those gathered under #characterbuildings and other hashtags related to housing are certainly a useful resource, it is important to note that there are limitations to these as a data source. To use the example of our study, an advantage of this research is capturing over 300 snapshots of rental housing experiences. However, given that the hashtag was promoted by a website focusing on news from the youth perspective, this is unlikely to be a representative sample of New Zealand Twitter users, or of New Zealanders. A further limitation is that, in contrast to data from surveys or in-depth interviews, tweets are directed at followers, and (if retweeted), the public. In this case, this public performance aspect may have been further amplified as there was a tacit understanding that *The Wireless* may also use the tweets in further articles on the topic. Drawing on Goffman’s [56] ideas of “impression management”, theorists have suggested that people carefully curate what they share on social media—a “selective disclosure of personal details designed to present an idealized self” [57]. Individuals using Twitter report that they tweet to a specific imagined audience, and are concerned with building a “personal brand” and gaining followers through their interactions with other users, including via the use of common hashtags [58]. It is impossible to know why people elected to use the hashtag, and why they chose a particular housing issue to report on, over other issues in their homes. It may be that people chose the most extreme or interesting housing experience to report on and wished to use it to draw attention to New Zealand’s housing issues and to align themselves with those seeking to challenge them. For these reasons, #characterbuildings, and perhaps Twitter more broadly, is useful for learning about how people communicate about housing conditions online, but is not an appropriate data source to learn about the distribution of housing conditions or even which housing conditions are of most concern to people using Twitter. Uneven access to the Internet and social media accounts across the population is also a clear limitation of this data. 

The impact of New Zealand’s housing on health is well-established [59,60,61]. While many tweets reported cold, damp and moldy housing conditions, which are likely to impact on health, only a few tweets referred specifically to health problems related to their housing. This may be partly because of how people “curate” their image on social media Research shows that although issues such as obesity or heart attacks are more common, people are much more likely to tweet about conditions like migraines and allergies, perhaps because these conditions are perceived of as being less embarrassing [62]; in the same way, people may feel more comfortable tweeting about housing experiences that have long passed, or that are less personal, which may explain why so few tweets in this example described housing-related health problems. Interestingly, the majority of comments about health were added with a sense of irony, which perhaps indicates that people living in substandard housing are so aware of health being a related problem that it is expected, in the same way that energy poverty is often described in New Zealand and also Australia as something to “put up” with as achieving adequate household temperatures in winter is often not possible [40,48]. 

This study has shown that qualitative analysis of Twitter data provides useful insight into people’s experiences, including what housing conditions they live in, their strategies to deal with housing problems, and how they portray these experiences in the public forum that is Twitter. Our analysis showed that people who shared stories using #characterbuildings added a human dimension to the statistics on housing quality, while also drawing attention to under-researched housing problems. Our analysis indicates that using social media may be particularly well-suited to capturing youth and younger-adult voices. Of the available platforms, Twitter is particularly useful for this, given that users tend to be younger than other social media users [11], though within our sample, a few nostalgic tweets reminiscing on previous residences indicated that some tweeters may have been older. Particularly in fuel poverty literature, the experiences of young people living in hard to heat and poor quality dwellings have been neglected [39,63,64,65]. 

This case of a media-instigated hashtag to promote a story provides a useful example of the extent to which social media may be used to generate new data, where pages or tweets are sufficiently followed, liked, or retweeted. The Wireless positions itself as providing “stories for New Zealanders who have grown up in the digital age” on both its Twitter and Facebook pages, where it has 7338 followers and 20,133 likes respectively as at 8 June 2017 [66,67]. Generation Rent, a UK-based advocacy organization, was able to generate similar stories about housing conditions through its Twitter and Tumblr campaigns #rantyourrent and #ventyourrent in the lead-up to the London Mayoral elections in 2016 [68]. Approaching such organizations to help by retweeting requests specifically for housing research—through, for example, asking followers to respond to “reply” to specific questions, or to share their thoughts or experiences using a particular hashtag—may be one way in which use of online tools can be used to access youth and younger adult voices that can be hard to reach through other recruitment strategies. 

Future researchers might also consider large-scale analysis of housing problems of Twitter users. Using large-scale analysis, Yin 2017 [62] was able to show what health issues people are most likely to report. Given the association between poor health and poor housing, it may be possible to build a similar housing index for use on Twitter and to look at whether there is an association between Twitter users who report certain health problems also report housing problems. In countries where housing quality is surveyed, future research could look at whether housing problems reported in geocoded tweets correlate with housing problems revealed in surveys. Such research has been undertaken looking at neighborhood satisfaction. Twitter analysis showed that tweets in particular locations were more likely to express sad emotions, but that this did not correlate with neighborhood satisfaction as expressed in the American Housing Survey [14]. 

The use of Twitter, and Twitter hashtags such as #characterbuildings, help spread awareness and build public support for particular issues: in this case, action on poor rental housing quality in New Zealand. This online discussion contributed to the national conversation about housing problems and the sense of collective outrage and need for urgency about addressing these problems. In the years following the #characterbuildings discussion, New Zealand’s first renters’ rights group was founded, renters’ voices were increasingly represented in the media, thousands of people participated in Park Up For Homes protests against homelessness, and the release of a People’s Review of Renting put pressure on government to take action. This collective action and social media advocacy helped opposition parties in New Zealand frame rental housing quality problems as part of a wider housing crisis, and saw housing become a key issue in the 2017 election, when a new Labour-led government was elected. This government plans to pass legislation to bring about higher standards for rental housing. The importance of social media in shaping how people think and what happens in the world means it is a key resource to shed light on issues of public and research concern.

The growth of Web 2.0 has broadened the role of the public health community engaging in media advocacy [69] to require online engagement for successful health promotion, and through allowing direct engagement with politicians and media in a public conversation can not only increase awareness but also help to shape or reframe public health issues [70]. We suggest that as well as in addition to online media advocacy, public health researchers should consider using social media to broaden the reach of recruitment and engagement of participants in studies allowing for new expressions of “voice” to be represented in housing research. Failure to do so may risk losing access to difficult to reach groups—including those who are more comfortable engaging through social media due to time poverty, privacy, or because online community engagement is the new normal for youth.

## 5. Conclusions

In this article, we have discussed the use of Twitter as a research tool for housing studies. We found that there is a dearth of research that takes advantage of this vastly populated online space. While interest appears to be growing, we argued that increasing the use of Twitter in public health and housing research either as a primary data collection tool, or as we have illustrated, through analyzing existing tweets, offers a new and useful avenue for obtaining rich information, particularly from a younger and often harder-to-reach demographic. 

We have presented empirical qualitative analysis of the Twitter media-initiated hashtag #characterbuildings as an example of using Twitter data to contribute to housing research. We found New Zealanders tweeting in response to #characterbuildings gave vivid examples of their struggles with inadequate housing conditions, particularly in the private rental sector. Analysis of this data offers storied examples of renters’ experiences, highlights previously unknown problems, for example slug infestations in damp or structurally deficient homes, and contributes to the discussion of problems with poor housing quality in New Zealand generally. 

Social media and Twitter provide useful data collection possibilities for public health and housing research. For studies in which timeliness, cost, carbon reduction, and inclusion of a younger demographic are key considerations in qualitative data collection, failing to engage with Twitter may allow this worthy data source to fly the coop. 

## Figures and Tables

**Table 1:** Categories of #Characterbuildings Tweets Identified.

Tweet Type	Exemplar	Frequency	95% CI
Structural issues	“Floor collapsed in laundry. Condensation dripping of ceiling in winter. Slugs. Black mold ceilings. Flaky asbestos”	103 34.7%	29.5–40.3%
Pests	Including rodents (20), slugs (10) and other insects (13). “I shared the hallway to my room with a family of slugs.”	43 14.5%	10.9–18.9%
Comments	“If you do not think the state of New Zealand’s rental properties is an issue that needs addressing, check out #characterbuildings”	43 14.5%	10.9–18.9%
Landlord	“Landlord put the rent up when I asked them to fix the waterfall that appeared in my room during heavy rain.”	37 12.5%	9.2–16.7%
Facilities	“Landlord came to fix toilet and just took cistern for a week, we flushed with a bucket and water from shower”	33 11.1%	8.0–15.2%
Mold	“Slugs coming through walls, bathroom ceiling covered in mold, couldn’t clean cos paint just peeled off, carpet rotting”	31 10.4%	7.5–14.4%
Damp	“would wake up in the morning in winter with wet duvet and walls from condensation”	28 9.4%	6.6–13.3%
Cold	“Colder temps inside in winter than outside. Shampoo froze in bottles.”	26 8.8%	6.0–12.5%
Costs	“Penthouse in our apartment building was leaky. Cue 1 year of concrete drilling 12/7 to sort it out. In addition, 2 rent rises.”	16 5.4%	3.3–8.6%
Layout	“Shower in the kitchen; good view from the bench if the curtain wasn’t quite shut”	15 5.1%	3.1–8.2%
Size	“My bathroom was so small, there was a bit cut out of the door so it could open across the toilet seat.”	8 2.7%	1.4–5.2%
Health	“Lived in probs [probably] the cheapest 4 brm flat in Palmy with 6 people—$25/week each. We paid the rest in respiratory illnesses.”	7 2.4%	1.1–4.8%
Flatmates	“Our flat was great until the flatty got bunnies that pooped inside, which lead to a maggot infestation.”	7 2.4%	1.1–4.8%
Neighbors	“The landlord (upstairs) would come home drunk and play loud music until 3 am. Her dog would shit in our backyard”	5 1.7%	0.7–3.8%
General/Other	This code captured all other issues raised only once, for example fire service commenting house was a death trap, having no letterbox, or having spotting knives melted into the bench.	18 6.1%	3.9–9.4%
